# MCM-41/PVA Composite as a Separator for Zinc–Air Batteries

**DOI:** 10.3390/ijms21197052

**Published:** 2020-09-25

**Authors:** Sirinuch Nanthapong, Soorathep Kheawhom, Chalida Klaysom

**Affiliations:** 1 Center of Excellence in Particle and Material Processing Technology, Department of Chemical Engineering, Chulalongkorn University, Bangkok 10330, Thailand; sirinuch.ntp@gmail.com; 2 Department of Chemical Engineering, Faculty of Engineering, Chulalongkorn University, Bangkok 10330, Thailand; soorathep.k@chula.ac.th; 3 Research Unit of Advanced Materials for Energy Storage, Chulalongkorn University, Bangkok 10330, Thailand

**Keywords:** zinc–air battery, composite membrane separator, composite gel polymer electrolyte, polyvinyl alcohol, MCM-41

## Abstract

Membrane separators are one of the critical components in zinc–air batteries (ZABs). In the control of mass transfer, and hence, electrochemical reaction, membrane separators have an important role to play. This work addresses the issue of battery performance in a ZAB via a new composite membrane separator based on polyvinyl alcohol (PVA). To enhance the electrolyte uptake and ionic conductivity, mesoporous Mobil Composition of Matter No. 41 (MCM-41) is incorporated as a filler in the membrane while maintaining its integrity. The presence of MCM-41 is seen to reduce the number of cycles of secondary ZABs due to the uninvited drawbacks of increased zincate crossover and reduced triple phase boundary at the air cathode, which is pivotal for oxygen reduction reaction. Overall, results suggest that the application of the MCM-41/PVA composite has the potential for use as a separator in high-capacity primary ZABs.

## 1. Introduction

Recently, zinc–air batteries (ZABs), being one of the relatively well-established metal–air batteries, has gained much attention because of their high potential as alternatives to lithium-ion batteries (LIBs) [1]. Zinc (Zn), used as an active material in ZABs, is abundant and cheap [2,3]. Compared to LIBs, the production cost of ZABs is, therefore, much lower and more sustainable [4]. Although the theoretical energy density of ZABs (~1086 Wh/kg) is estimated to be five times higher than that of existing LIBs, commercialization of ZABs is hindered by several technical issues. These challenges are mostly related to component materials such as electrodes, electrolyte, and separator [5]. However, great strides have been made in the development of novel zinc electrodes and battery design [6,7,8], electrolyte additives [7,9,10], oxygen reduction, and evolution electrocatalysts [11,12,13]. Nevertheless, in comparison to other components, the separator, which is a key component for controlling the mass transport of electrochemical reactions, has not yet received its deserved attention.

The primary function of a membrane separator is to prevent a short circuit from direct contact between the anode and the cathode while selectively facilitating the transport of active species such as hydroxide ions and water molecules to maintain stable charge–discharge cycles [14]. The ideal membrane separator, therefore, must possess not only high electrolyte uptake, high electrical resistance, and excellent ionic conductivity, but should also be chemically stable, even in a strong alkaline electrolyte. In addition, it should be able to prevent the crossing over of zincate ions from the zinc electrode to the air electrode. The crossing over of zincate ions brings about an increase in battery polarization and loss of performance over time [15]. To obtain all the desired properties for the membrane separator abovementioned, careful material design and optimization must be executed.

Hydrophilic polymers like polyvinyl alcohol (PVA) [16], polyvinyl acetate (PVAc) [17], and polyethylene oxide (PEO) [18] are typically used for making a polymer gel electrolyte (PGE) membrane. Of these polymers, PVA is the most widely used material, as a PGE separator for ZABs. PVA stimulates extensive swelling in water and provides high electrolyte uptake and excellent ionic conductivity. However, extensive swelling reduces its mechanical stability. Moreover, PVA is not stable in excess alkaline electrolytes [19,20,21]. In addition, adhesion between a pure PVA separator and the electrodes is proved to be quite poor and can lead to unstable discharge voltage of ZABs. Therefore, using pure PVA is not the best option in this application.

One essential property for the membrane separator is to hold up high electrolytes (electrolyte uptake) to perform the electrochemical reaction with a zinc electrode. Typically, the strategies to enhance the membrane electrolyte uptake involve controlling the structure and microporosity of the membrane [22,23] or the use of highly hydrophilic polymers that can swell aqueous electrolytes well [14,20]. However, the main disadvantage of using porous membranes is the migration of other ions besides the electrolytes and the evaporation of liquid electrolytes from the pores that result in unstable battery performance and short rechargeable cycle. On the other hand, the main setback for using a swollen electrolyte polymer is its inevitably poor mechanical stability.

One approach to enhance membrane stability and mechanical properties is to combine an inorganic filler into a polymer matrix [24]. It is acknowledged that combining inorganic fillers to form a polymer matrix composite (PMC) can improve the overall performance in many membrane applications. These applications include fuel cell [25], ion-exchange membranes in desalination [26], and redox flow battery [27,28]. They are closely related applications for ZABs. Besides, similar schemes have also been proved for developing a membrane separator for ZABs. For instance, mesoporous SiO_2_ nanoparticles have been functionalized with sulfonic acid and applied as an additive in a polymer matrix [29]. Thereby, it was reported that a small addition of the sulfonated nanoparticle could improve water uptake, ionic conductivity, and ion-exchange performance of the membrane in desalination via an electrodialysis process. Wang et al. [30] found that mesoporous silica added to polyvinylidene fluoride-hexafluoropropylene (PVDF-HFP)-based gel electrolytes formed a strong silica network with PGE for LIBs. The composite gel electrolyte exhibited not only enhanced ionic conductivity but also better mechanical stability. High discharge capacity over 130 mAh/g and high coulombic efficiency (>90%) was achieved. Enhanced electrochemical properties of a solid polymer electrolyte has also been observed when ceramic zirconium oxide (ZrO_2_) nanoparticles have been added to PVA. The inorganic filler nanoparticles acted as a plasticizer for the polymer, increasing free volume in the parent polymer that promoted the transport of the electrolyte [31].

In 1992, MCM-41, a mesoporous material, was introduced by the Mobil Research and Development Corporation [32]. MCM-41 has a large specific surface area of 1000 m^2^/g and pore volume of 1 cm^3^/g with a very narrow pore size distribution. In addition, MCM-41 shows adjustable hydrophobicity, an ability to modify surface functionality, and has very good thermal stability. Saputra et al. [33] used MCM-41 directly as an inorganic separator for ZABs and achieved a stable discharge voltage at 1.2 V. However, the discharge capacity of the battery was found to be very low when compared to other PGE membranes [22]. This might be because MCM-41 cannot adsorb and hold up as much alkaline electrolyte as a polymer. In this work, MCM-41 is employed as a filler for PVA, forming a novel composite gel polyelectrolyte membrane for both primary and secondary ZABs. The effects of MCM-41 loading on the properties and performance of the membrane separator in ZABs are duly investigated.

## 2. Results & Discussion

### 2.1. Effect of Filler Loading on Membrane Property

**Figure 1:**
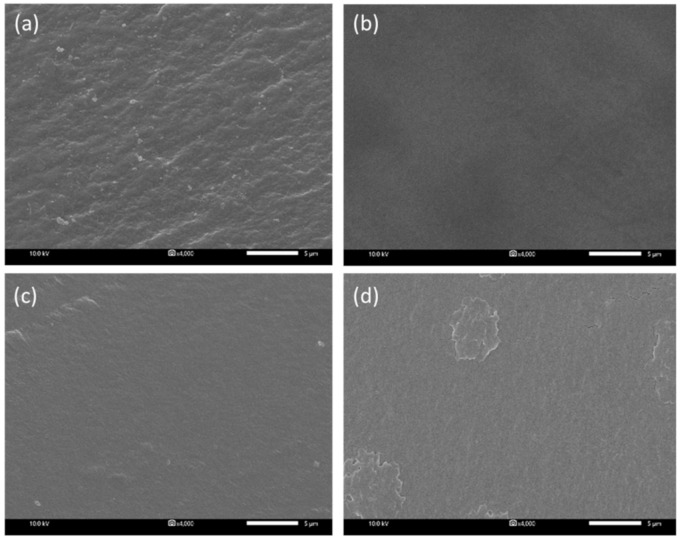
SEM images of cross-section area: (**a**) PVA membrane without MCM-41, (**b**) PVA membrane containing 2.5 wt.% MCM-41, (**c**) PVA membrane containing 5 wt.% MCM-41, and (**d**) PVA membrane containing 7.5 wt.% MCM-41.

Different amounts of MCM-41 particles were added to the PVA to form the composite membrane separator. The effect of the amount of filler on the membrane structure was investigated by a scanning electron microscope (SEM) (Figure 1). The structure of all the prepared membranes was dense. It is a typical structure obtained via a phase inversion using the solvent evaporation method.

**Figure 2:**
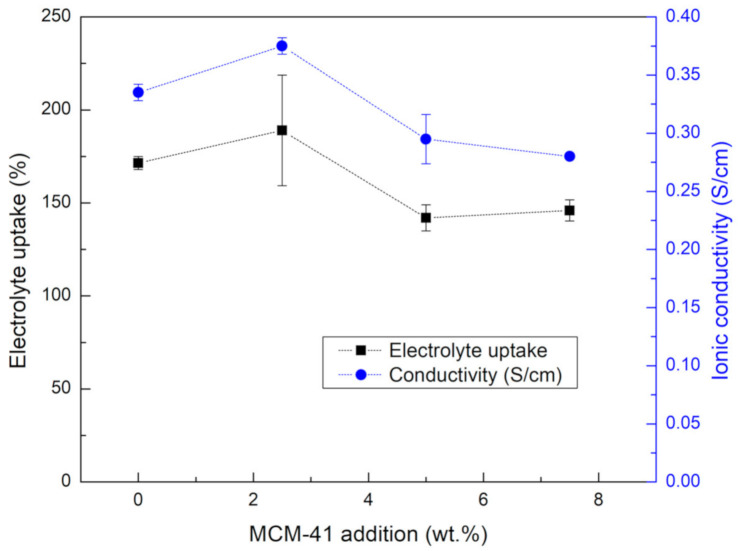
The electrolyte uptake and ionic conductivity of a composite MCM-41/PVA membrane with different amounts of MCM-41 loading.

It is believed that a membrane separator possessing high electrolyte uptake and high amorphous phase could promote the transportation of hydroxide ions and battery performance [34]. There are several strategies to enhance the ionic conductivity of polymer membrane, such as blending with a high hydrophilic polymer, adopting modifiers, and incorporating inorganic filler [34]. The addition of inorganic filler into a polymer matrix was reported to be a promising approach. The inorganic filler can serve as a plasticizer for the polymer, enhancing the segmental motion of the polymer that increases free volume, reduces the crystallization of polymer, and thus promotes transport of ions to the electrochemical reaction region. The electrolyte uptake and ionic conductivity of the composite MCM-41/PVA membrane having different amounts of MCM-41 loading were investigated and are illustrated in Figure 2. In this work, a small loading of MCM-41 up to 2.5 wt.% was found to enhance both electrolyte uptake as well as ionic conductivity of the membranes. However, at a higher loading (5–7.5 wt.% of MCM-41), electrolyte uptake and ionic conductivity tended to decrease. Apparently, a small loading of inorganic nanoparticles can interrupt the packing of the polymer chain and increase the free volume of the matrix polymer that enhanced the uptake capacity of the electrolyte and ionic conductivity [35]. Likewise, excessive filler loading (>2.5 wt.%) can result in material phase separation due to the agglomeration of the fillers. Such observations of the critical threshold of filler loadings are commonly reported for many composite materials [26,36]. Nevertheless, in this work, the electrolyte uptake and ionic conductivity of all prepared membranes are relatively high compared to those reported in the literature based on the similar polymer [20,22,23].

### 2.2. Primary Battery Performance

**Figure 3:**
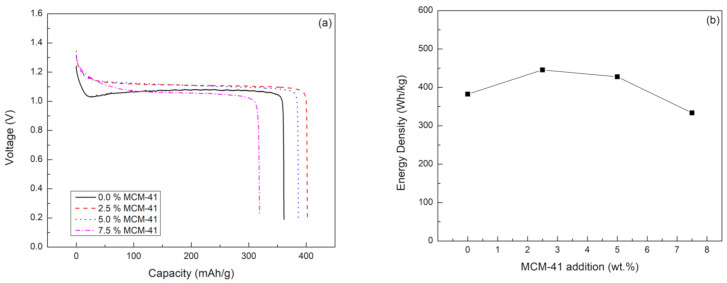
(**a**) Discharge capacity and (**b**) energy density of ZABs using a composite MCM-41/PVA membrane having different amounts of MCM-41 loading at constant discharge current of 5 mA.

The performance of the prepared coin cell battery with different membrane separators was investigated. Figure 3 shows discharge profiles and energy densities of the batteries as a function of MCM-41 loading. Consequently, the batteries with the composite separators having 2.5 and 5 wt.% MCM-41 loading showed stable discharge voltage at around 1.11 V. However, the battery with the composite separator having 7.5 wt.% MCM-41 and the battery using PVA without MCM-41 exhibited lower discharge capacity and displayed lower discharge voltage at around 1.05 and 1.07 V, respectively. The higher discharge voltage of the composite separator may be due to higher electrolyte uptake, better ionic conductivity, and better interface contact between separator and electrode. The composite separator, having a higher electrolyte swollen degree, can form a softer structure and generate good adhesion with the electrodes. It is worth noting that the trend of battery performance in terms of discharge capacity and energy density is reflected in the trend of electrolyte uptake of the prepared membranes having different loadings. Thus, the ZAB using the composite membrane containing 2.5 wt.% MCM-41 in PVA demonstrated the highest performance, having a discharge capacity of 400 mAh/g and energy density of 446 mWh/g. The discharge voltage (1.05–1.11 V) of the battery using PGE developed in this work is comparable to other types of membrane separator or PGE reported in the literature [22,23,33].

### 2.3. Rechargeability of ZABs

**Figure 4:**
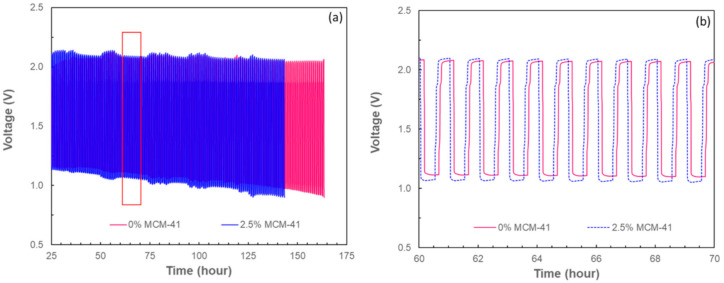
(**a**) The relation between charge–discharge voltage (V) and time (h) of rechargeable ZABs using PVA without MCM-41 and 0.25 wt.% MCM-41/PVA composite membranes and (**b**) its expanded view at 60–70 cycle time (the region marked in red box in (**b**)).

The cyclability of rechargeable ZABs using the selected membranes (the PVA without MCM-41 and the composite with 2.5 wt.% of MCM-41) was also examined. Figure 4 compares the charge–discharge voltage of rechargeable ZABs having different membranes. The charge voltage of both batteries was found to be similar and constant at around 2.1 V, though their discharge voltage gradually decreased over time. However, a gradual increase in voltage gap is seen between charge and discharge state of both batteries. It is feasible that the increase in voltage gap could be due to several coexisting effects such as water loss, ionic conductivity degradation, and the development of resistance layer from zincate crossover. In the case of the battery with the composite membrane, the voltage gap increased faster, resulting in a shorter battery life cycle (around 145 cycles for the composite membrane). In the case of the battery with PVA membrane without MCM-41, 163 cycles were achieved.

Subsequently, to check out what contributed to the shorter battery cycle of the composite separator, both resistance build-up over time and zincate crossover of the membranes were examined.

**Figure 5:**
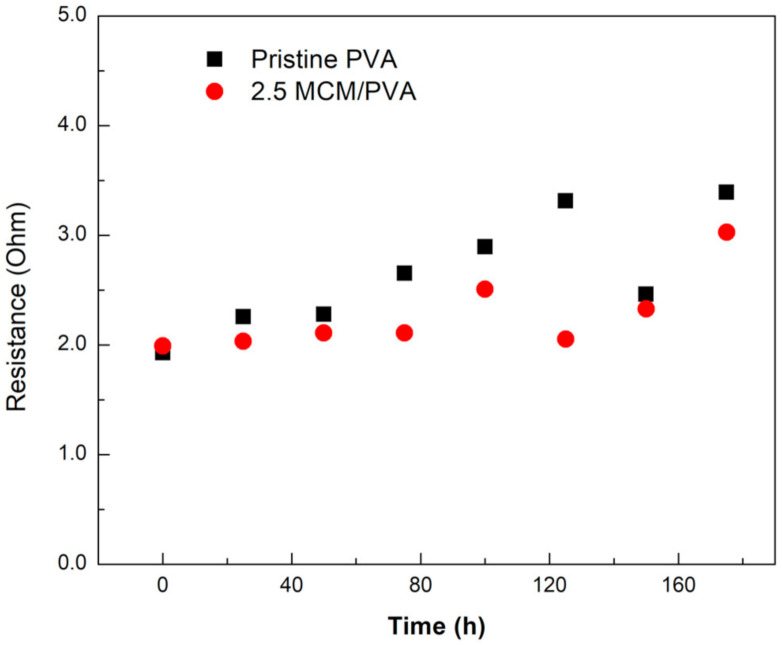
The resistance buildup of rechargeable ZABs having different composite membranes.

**Figure 6:**
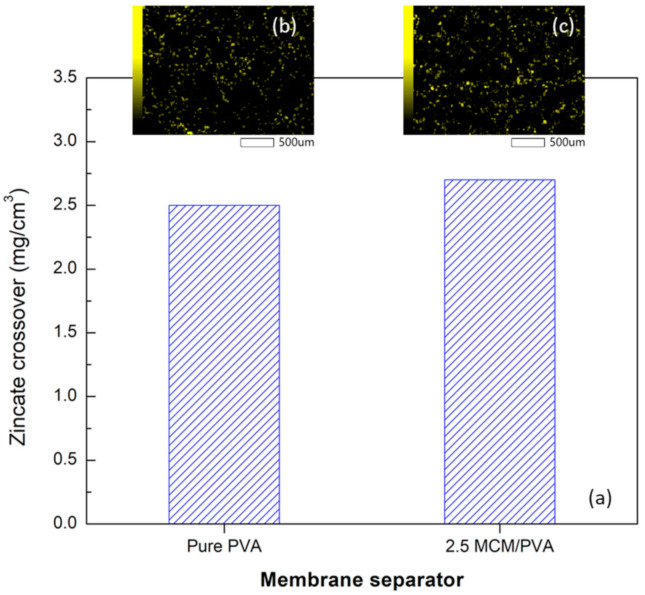
(**a**) Zincate crossover of the PVA membrane without MCM-41 and 2.5 wt.% MCM/PVA composite membrane after 150 h of use (**b**) and (**c**) EDS element mapping of Zn on the air cathode in contact with the PVA membrane and the composite membrane, respectively.

Figure 5 and Figure 6 the resistance buildup and zincate crossover of the PVA without MCM-41 and the composite membrane. Initially, the resistances of both battery cells were seen to be similar (Figure 5). Once the batteries started cycling, the resistance of the cells gradually built up. The resistance of the battery cell having a PVA membrane without MCM-41 seemed to increase at a rather faster rate than that of the 2.5 wt.% MCM/PVA. From Figure 6, the zincate crossover, i.e., 2.5 wt.% MCM-41/PVA composite membrane was found to be slightly larger than the PVA membrane without MCM-41. The deposition of ZnO on the air cathode in contact with the membrane from energy-dispersive X-ray spectroscopy (EDS) revealed the deposition of Zn atoms (yellow spots in Figure 6b,c). Both cases were comparable. The unexpected shorter battery cycle in the case of the battery having the composite membrane might be attributed to the passivation layer from ZnO precipitation on the air cathode which came about from a higher zincate crossover. In addition, it might also be attributed to water loss and change in dimension of the membrane, leading to an integrity problem between membrane separator and air cathode. It is worth noting here that the rechargeable cell used in this experiment is in a different configuration to the primary coin cell in which one side of the membrane was exposed to the electrolyte and the other side was attached to the air electrode and ambient air. This testing configuration poses even more challenges for a membrane to retain its dimensional stability, especially for a membrane with high water or electrolyte uptake. Such a set-up could cause a problem around the interface of the membrane and cathode, inevitably degrading the battery discharge and rechargeable cycle. Although having a higher electrolyte uptake, the battery with the composite membrane seemed to have little ability to retain water or its shape. Thus, this deficiency led to faster degradation of discharge voltage and a shorter battery life cycle. Though the microstructure and characterization of the sample after the experiment is essential for a better understanding about the degradation mechanism of the material, it is difficult to disassemble the battery cell after the battery tests without damaging each layer. The damage from sample handling could mislead the results. In situ characterization techniques are required and recommended for future work. To further improve the mechanical properties of the membrane, we suggested applying some crosslinking agents such as a silane coupling agent [34]. However, careful control for the crosslinking must be exercised to not over-crosslink and jeopardize the electrolyte uptake and ionic conductivity of the membrane.

As summarized in Table 1, the properties and performance of the prepared membrane separator in ZABs were compared with other works. However, there is a limit to the number of publications reporting rechargeable battery performance of ZABs assembled from newly developed membranes. In addition, it is significant that the electrochemical properties of materials are highly dependent on the testing conditions. Therefore, it is not an easy task to compare results from different research studies. Nevertheless, the batteries used in this present study having the prepared membranes demonstrated outstanding performance, exhibiting high energy density and battery life cycle. In general, this was due to the high electrolyte uptake and ionic conductivity of the prepared membranes.

## 3. Materials and Methods

### 3.1. Materials

Tetraethylorthosilicate (TEOS, 99%+) was purchased from Sigma–Aldrich (Steinheim, Germany). Polyvinyl alcohol (PVA, MW 100,000 Da) was obtained from Chem-supply (Gillman, South Australia, Australia). Cetyltrimethylammonium bromide (C_16_TAB, 98%) and potassium hydroxide (KOH, Pellets) were purchased from Asia Pacific Specialty Chemical Limited (Cherrybrook, New South Wales, Australia). Ammonia solution (NH_3_, 25%) was obtained from QREC (New Zealand). All chemicals were used as received.

### 3.2. Synthesis of MCM-41

MCM-41 was prepared via a modified Stöber method [37], using TEOS as the silica source, C_16_TAB as the surfactant, ammonia solution (NH_3_) as the agent for silicate condensation, and deionized (DI) water as the solvent. In a typical synthesis, the molar ratio of C_16_TAB:NH_3_:TEOS:DI water was controlled at 1:0.3:11:144. Firstly, C_16_TAB, NH_3_, and TEOS were mixed in DI water and stirred at room temperature for 2 h. Thus, the precipitated white powder of MCM-41 was filtered out and washed with DI water. Then, the obtained powder was dried at 100 °C overnight in an oven and calcined in the air to remove all residual chemicals at 550 °C for 6 h.

### 3.3. Preparation of the Membrane Separator

Firstly, PVA polymer was dissolved in DI water to a fixed total polymer concentration of 13 wt.%. A desired amount of MCM-41 was added to the solution of polymer. The solution was then heated at 80 °C under stirring until homogeneous. Next, 6 M KOH (50 wt.%) was poured into the solution and mixed together. The prepared solution was again poured into a petri dish with a controlled thickness of 1.8–2.0 mm and air-dried at room temperature overnight. The prepared membrane was immersed in 6M KOH (alkaline electrolyte) at room temperature for 24 h before use.

### 3.4. Material Characterization

Crystallization of the synthesized MCM-41 was obtained via X-ray diffraction (XRD) (BRUKER, D8 Advance (λ = 1.5406 Å). The structure of the synthesized MCM-41 was observed by a transmission electron microscope (TEM, JEOL, TEM-3100F, Tokyo, Japan). The characteristics of the prepared MCM-41 fillers are provided in the supplementary information (Figure S1).

The morphology of the prepared membrane was observed by a scanning electron microscope (SEM, JEOL, JSM-IT500HR, Tokyo, Japan). The electrolyte uptake of membranes was estimated by measuring the weight change of the membrane before and after immersion in an electrolyte, as in Equation (1):

(1) $$\mathrm{Electrolyte}\;\mathrm{uptake}\;\left(\%\right)=\frac{W_{\mathrm{wet}}\;-\;W_{d\mathrm{ry}}}{W_{\mathrm{dry}}}\times 100$$

where *W*_wet_ is the weight of the composite membrane after soaking in 6 M KOH aqueous solution at room temperature for 24 h and *W*_dry_ is the dry weight of the prepared membrane before being soaked in 6 M KOH aqueous solution.

Ionic conductivity of the prepared membranes was evaluated by electrochemical impedance spectroscopy (EIS) in the frequency range 0.15 Hz–150 kHz. All samples were immersed in 6 M KOH at room temperature for 24 h before the test. Ionic conductivity (σ, mS/cm) of the membrane was calculated, as shown in Equation (2):

(2) $$\sigma =\frac{L}{R_{b}A}$$

where *L* is the membrane thickness (cm). *R_b_* is the membrane resistance (Ω), which can be estimated from the Nyquist plot obtained from the EIS. *A* is the area of the membrane sample (cm^2^).

**Figure 7:**
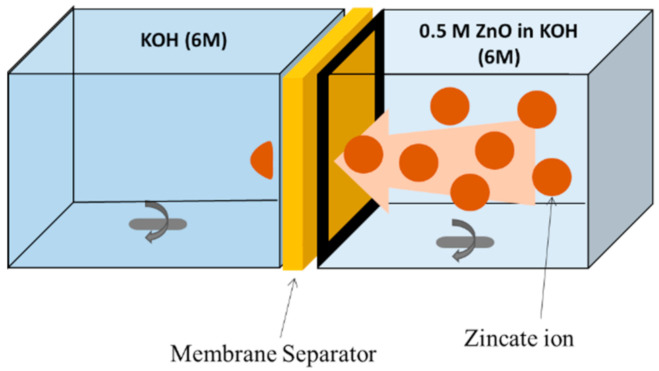
Schematic of the two-compartment cell used for measuring zincate crossover

Following the procedure previously reported [22], the crossover of zincate ions (Zn(OH)_4_^2^^−^) through the membrane was investigated using a two-compartment cell, as illustrated in Figure 7. As shown, a membrane sample was placed between the two compartments. One compartment was filled with 6 M KOH aqueous solution, simulating the electrolyte solution used in all battery tests. The other contained 0.5 M zinc oxide (ZnO in 6 M KOH), simulating the presence of zincate ions in the system. The zinc oxide concentration difference between the two channels created a driving force for the zincate ions to diffuse through the membrane. The solution in both compartments was stirred at room temperature to ensure uniform mixing. The change in ZnO concentration was measured by an inductively coupled plasma (ICP, Agilent, ICP-OES 5100, Santa Clara, CA, USA) and the transport (diffusion) of zincate ions from one side to the other was recorded.

### 3.5. ZAB Fabrication and Performance Test

**Figure 8:**
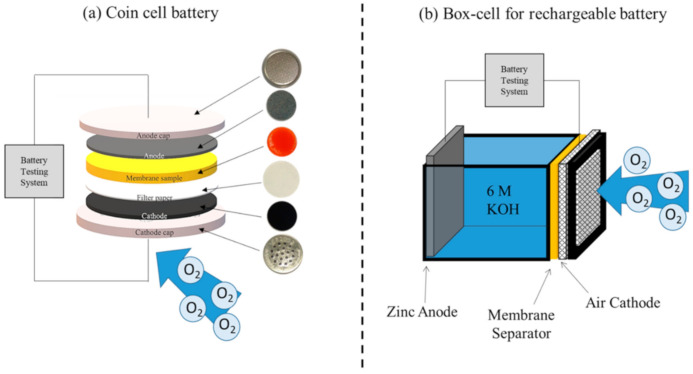
Schematic view of zinc–air battery (ZAB) in (**a**) primary cell and (**b**) secondary cell configuration.

The performance of the membrane separator used as a separator, in both primary and secondary ZABs, was investigated using battery testing system (NEWARE, BTS7.6.0, Shenzhen, China). Figure 8 shows the schematic view of the coin cell and the rechargeable cell (box cell) used in this work.

The primary ZAB was fabricated as a coin cell (CR2032) and was comprised of an air cathode (0.785 cm^2^), a zinc anode (0.785 cm^2^), and a composite membrane separator/electrolyte (0.785 cm^2^). Similarly, the rechargeable cell consisted of an air cathode (1 cm^2^), a zinc anode (1.5 cm^2^), a composite membrane separator (1 cm^2^), and 6 M KOH aqueous electrolyte. The zinc anode was formed using an electroplating technique, whereby Zn was electrodeposited from 1 M zinc sulfate solution on nickel (Ni) foam (4 × 4 cm^2^) under an applied current density, i.e., 25 mA/cm^2^ for 6 h. The air cathode was fabricated by firstly coating it with a slurry of PTFE, carbon (C), glucose, and sodium bicarbonate (NaHCO_3_) at a 1:1:0.25:0.25 weight ratio (in ethanol) on one side of the Ni foam. The coated Ni foam was dried at 350 °C in an oven to form a gas diffusion layer. Then, the catalyst layer was fabricated using a mixture of C and MnO_2_ at a 1:1 weight ratio (dissolved in 3 wt.% PVDF in toluene). The mixture was coated on the other side of the Ni foam and then dried in an oven at 70 °C for 20 min. The coated Ni foam was then further compressed using a rolling mill. For the primary cell, the membrane separator soaked with 6 M KOH was placed between the air cathode and the zinc anode. The performance of the primary coin cell was evaluated using a galvanostatic technique at a controlled discharge current of 5 mA. Discharge voltage was recorded. The discharge capacity, indicating total available capacity per unit mass of zinc anode used, was then estimated, according to Equation (3):

(3) $$\mathrm{Disharge}\;\mathrm{capacity}=\;\frac{\mathrm{Capacity}\;\left(\mathrm{mAh}\right)}{\mathrm{Weight}\;\mathrm{of}\;\mathrm{zinc}\;\left(g\right)}$$

where energy density, representing the discharge energy calculated by multiplying the discharge power (W) by the discharge time (h) per mass of Zn anode used, is expressed as follows in Equation (4):

(4) $$\mathrm{Energy}\;\mathrm{density}=\;\frac{\mathrm{Energy}\;\left(\mathrm{mWh}\right)}{\mathrm{Weight}\;\mathrm{of}\;\mathrm{zinc}\;\left(g\right)}$$

The performance of the rechargeable ZAB was evaluated using the box cell battery filled with 10 mL of 6 M KOH (Figure 8b). The rechargeable performance (cycle number) of the prepared battery was carried out at room temperature having a controlled charge–discharge current of 5 mA and discharge capacity of 2.5 mAh. The cell was repeatedly discharged and charged; with each cycle period being 30 min discharge and 30 min charge. A battery tester was programmed to immediately terminate the battery cell if it reached either charge or discharge cut-off voltage at 2.4 and 0.9 V, respectively. In order to observe the zincate crossover, after the cycling test, the deposition of ZnO on the air cathode, which was in contact with the membrane, was examined via SEM and energy-dispersive X-ray spectroscopy (EDS; Oxford, X-MaxN20, Abingdon, UK).

It is worth remarking here that 6 M KOH was used as the alkaline electrolyte in all characterization and battery tests. At this concentration, the electrolyte provided a high content of hydroxide ions for the electrochemical reaction, but not too viscous for the ionic to transport through [38]. It was also reported to be in the range of suitable alkaline electrolyte concentration for ZABs in the literature [38,39,40].

## 4. Conclusions

Herein, a new composite membrane separator based on PVA was developed. Thus, mesoporous MCM-41 was used as a filler for the membrane separator. The effect of filler loading on the physical and electrochemical properties of the membranes was investigated. A small loading of MCM-41 (up to 2.5 wt.%) was found to improve both electrolyte uptake and ionic conductivity of the membranes. Hence, both discharge capacity and energy capacity of the battery assembled with the composite membrane increased over 40%. The concept of incorporating inorganic material such as MCM-41 in PGE proved useful for enhancing the electrochemical properties of the membranes. It is evident that the battery life cycle can be further prolonged, if the mesoporous inorganic fillers that proved to be stable in the strong alkaline electrolyte and retarded zincate crossover are implemented.

## Figures and Tables

**Table 1:** Electrochemical properties and battery performance of membranes currently developed for ZABs.

Membrane Separator/Electrolyte	Electrochemical Properties		Battery Performance			Ref.
	Electrolyte Uptake (%)	Ionic Conductivity (mS/cm)	Discharge Capacity (mAh/g)	Energy Density (mWh/g)	Rechargeable Cycle (Testing Conditions)	
PVA	118	13.5	N/A	N/A	N/A	[22]
PVA on PEI nanomat support	87	13.1	645	N/A	7 (N/A)	[22]
PVA/PAA nanomat	31.2	6.6	N/A	N/A	250 (at 20 mAcm^−^^2^, 10 min/cycle)	[23]
PVA/PAA	61.1	11.2	N/A	N/A	75 (at 20 mAcm^−^^2^, 10 min/cycle)	[23]
PVA-KOH	N/A	N/A	N/A	N/A	37.5 (at 3 mA cm^−^^2^, 20 min/cycle)	[16]
PVA	172	340	385	382	163 (at 5 mAcm^−^^2^, 1 h/cycle)	This work
2.5 MCM-41/PVA	169	380	400	446	145 (at 5 mAcm^−^^2^, 1 h/cycle)	This work

## Supplementary Materials

The following are available online at https://www.mdpi.com/1422-0067/21/19/7052/s1, Figure S1. (**a**) TEM image and (**b**) XRD patterns of the synthesized MCM-41.

## Abbreviations

ZAB	zinc–air battery
PVA	polyvinyl alcohol
MCM-41	Mobil Composition of Matter No. 41
LIB	lithium-ion battery
PVAc	polyvinyl acetate
PEO	polyethylene oxide
PGE	polymer gel electrolyte
PVDF	polyvinylidene fluoride
PVDF-HFP	polyvinylidene fluoride-hexafluoropropylene
PTFE	polytetrafluoroethylene
TEOS	tetraethylorthosilicate
XRD	X-ray diffraction
SEM	scanning electron microscope
EIS	electrochemical impedance spectroscopy
EDS	energy-dispersive X-ray spectroscopy
ICP	inductively coupled plasma
