# Supplementary material for: MCM-41/PVA Composite as a Separator for Zinc–Air Batteries
Source: Int J Mol Sci. 2020 Sep 25;21(19):7052. doi: 10.3390/ijms21197052 (PMC7582340; doi:10.3390/ijms21197052)
Supplement: Supplementary file 1 [file ijms-21-07052-s001.pdf]

MCM-41/PVA Composite as a Separator for Zinc-Air Batteries

MCM – 41 was successfully synthesized. The structure of the MCM-41 was observed by using TEM and illustrated in Figure 3(a). It indicated cylindrical pores typically found in MCM-41. In addition, the XRD pattern in Figure 3 (b) indicates a narrow reflection peak at 2.3° and less pronounced peaks at 4.9° and 4.1° that confirms the characteristics of mesoporous MCM-41. In addition, the surface area of the MCM-41 was measured using N_2 adsorption/desorption isotherm and was calculated to be 1651 m^2/g with a mean pore diameter of 2.38 nm.

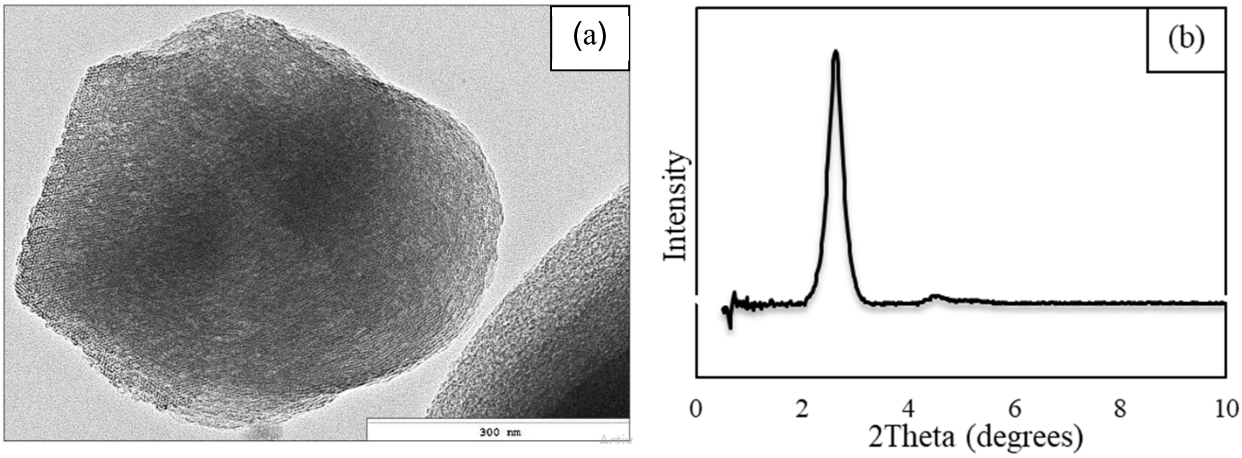

Figure S1. (a) TEM image and (b) XRD patterns of the synthesized MCM-41.
